# A Protective Mechanism against Antibiotic-Induced Ototoxicity: Role of Prestin

**DOI:** 10.1371/journal.pone.0017322

**Published:** 2011-02-25

**Authors:** Li Yu, Xiao Hua Jiang, Zhen Zhou, Lai Ling Tsang, Mei Kuen Yu, Yiu Wa Chung, Xiao Hu Zhang, Ai Mei Wang, Hao Tang, Hsiao Chang Chan

**Affiliations:** 1 Epithelial Cells Biology Research Center, School of Biomedical Sciences, Faculty of Medicine, The Chinese University of Hong Kong, ShaTin, Hong Kong; 2 Department of Physiology, Liao Ning Medical University, Jin Zhou, Liao Ning, China; 3 Department of Physiology, China Medical University, Shen Yang, Liao Ning, China; Charité-University Medicine Berlin, Germany

## Abstract

Hearing loss or ototoxicity is one of the major side effects associated with the use of the antibiotics, particularly aminoglycosides (AGs), which are the most commonly used antibiotics worldwide. However, the molecular and cellular events involved in the antibiotic-induced ototoxicity remains unclear. In the present study, we test the possibility that prestin, the motor protein specifically expressed in the basolateral membrane of outer hair cells (OHCs) in the cochlea with electromotility responsible for sound amplification, may be involved in the process of AG-induced apoptosis in OHCs. Our results from both mice model and cultured cell line indicate a previously unexpected role of prestin, in mediating antibiotic-induced apoptosis, the effect of which is associated with its anion-transporting capacity. The observed downregulation of prestin mRNA prior to detectable apoptosis in OHCs and hearing loss in the antibiotic-treated mice is interesting, which may serve as a protective mechanism against hearing loss induced by AGs in the early stage.

## Introduction

Despite their well known side effects such as ototoxicity and nephrotoxicity, aminoglycosides (AGs) are still the most commonly used antibiotics world- wide due to their high efficacy and low cost. However, their extensive usage in developing countries recent years have resulted in significantly higher incidences of drug toxicity, which could be a result of increased application in multidrug-resistant tuberculosis requiring long-term therapy, over the counter availability and poor monitoring of auditory function following over-course treatment [1]. The incidence of AGs-associated hearing loss reported ranges from a few percent up to 33% and is projected to afflict more people worldwide in the next twenty years due to the outbreak of drug-resistant tuberculosis in developing countries [2].

All AGs have the potential to induce severe and irreversible ototoxicity; however, the mechanisms underlying AGs-induced hearing loss or ototoxicity remain unclear. Prolonged exposure of the cochlear cells to AGs is apparently linked to the damage of the outer hair cells (OHCs) in the organ of Corti, leading to permanent hair cell loss and hearing damage [3], [4]. Many studies have indicated that generation of free radicals, abnormal iron transport, dysfunction of mitochondria, gene mutations and drug-drug interactions are involved in ototoxicity induced by AGs [5], [6], [7], [8], [9], [10]. These cellular events can initiate several different mechanisms of cell death depending on the type of AGs exposure. While necrotic death has been observed in animal models for decades, numerous studies have also shown that AGs-induced apoptosis in OHC is responsible for the drug-induced hearing damage [3], [11], [12], [13].

OHCs in the mammalian cochlea, apart from being the sensory unit, also generate force to amplify sound-induced displacements of the basilar membrane thus enhancing auditory sensitivity and frequency selectivity. This force generation is attributed to the voltage-dependent contractility of the OHCs underpinned by the motor protein, prestin. Prestin, a member of the SLC26 anion transporter family, is located at the basolateral wall of OHCs and responsible for their voltage-driven electromotility[14]. Early studies failed to demonstrate ion transporting capacity of prestin, unlike its nonmammalian orthologs and mammalian family members. It has been suggested that the prestin's voltage-sensing capacity requires an incomplete transport cycle which depends on its binding and hemimovement of anions within the intramembranous protein [15], [16]. However, a recent study demonstrated that prestin was able to transport anions, such as chloride or bicarbonate, across the membrane, which could act as voltage sensor localized at the cytoplasmic side of the membrane [17]. Given that OHCs, which specifically express prestin, are prone to undergo apoptosis in response to AGs, it is tempting for us to speculate and test the possibility that prestin may be involved in the process of AGs-induced apoptosis in OHCs.

## Results

### Chronic kanamycin treatment leads to hearing and OHC damage

**Figure 1:**
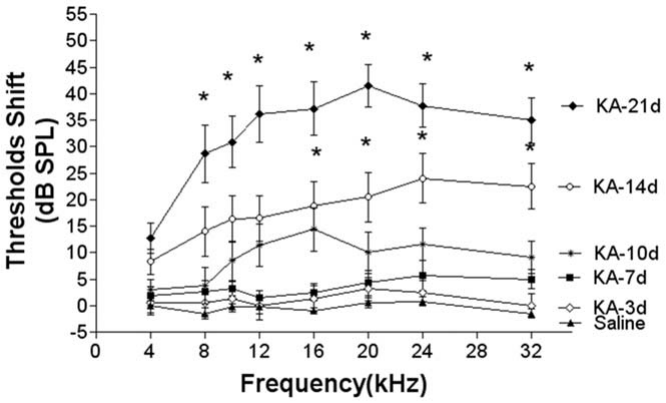
Threshold shifts in mice treated with kanamycin. BALB/c male mice, 4 weeks old, 10 each group, were administrated with kanamycin at 750 mg/kg with subcutaneous injection twice daily. Saline injection was served as a control. Thresholds of ABR were measured with tone-pip pure tone at 4 k, 8 k, 12 k, 16 k, 20 k, 24 k, and 32 k Hz. Thresholds of hearing shifted upward in a time-dependent manner after 14 days of treatment.

Mice were subcutaneously injected with kanamycin (750 mg/kg, twice daily) for different periods of time and hearing loss was assessed by measuring thresholds in auditory brain stem responses (ABR). As shown in Figure 1, saline-injected mice maintained stable hearing response at any frequency tested during treatment. There were no significant changes in ABR thresholds after 7 days of kanamycin treatment; however, continued injection of kanamycin for 14 days resulted in a frequency-dependent threshold shift. In addition, consecutive injection for 21 days led to significant threshold shifts at all frequencies tested, with the largest threshold shift of about 35–40 dB peaked at 20 kHz (Fig. 1).

**Figure 2:**
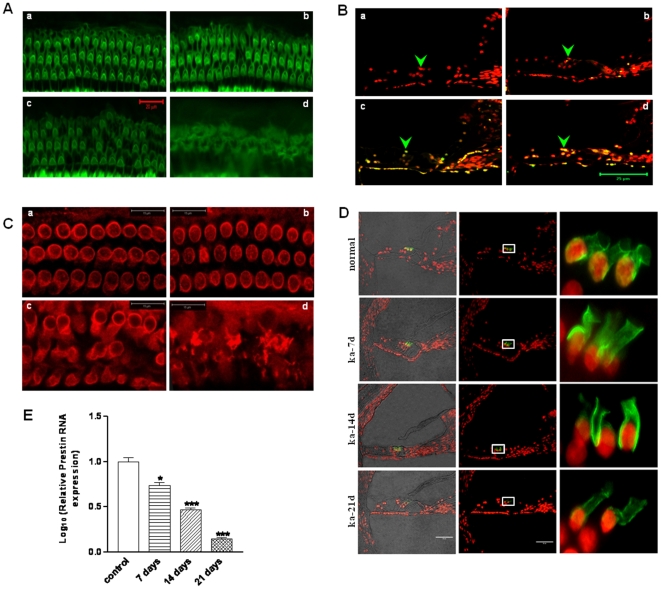
Kanamycin induces hearing damage, apoptosis and down-regulation of prestin in OHC. 4 week old BALB/c male mice were injected with 750 mg/kg kanamycin subcutaneously twice daily for the time indicated. (**A**): FITC-conjugated phalloidin (green) staining on cross section of OHC. a, normal control; b, ka-7d; c, ka-14d; d, ka-21d. Scale bar = 20 µm. (**B**): TUNEL assay of cochlear sections. Cochlear sections were stained with TUNEL (green) and propidium iodide (red). a, Normal morphology and no TUNEL-positive staining was found in inner ear cells in saline-treated control animals; b–d, 7, 14 and 21 days of kanamycin treatment. The sections shown are representative of three individual animals at each time point. Scale bar = 25 µm. Green arrow head: OHC cells. (**C**): Rhodamine-conjugated prestin staining on cross section of OHC. a, Normal, normal staining of prestin with three rows of rings; b, ka-7d; c, ka-14d; d, ka-21d. Scale bar = 15 µm. (**D**): Prestin immunofluorescence staining of cochlear sections. Cochlear sections of control, 7, 14 and 21 days after kanamycin treatment were stained with prestin at 1∶100 dilutions. Scale bar = 50 µm. Left column: immunostaining merged with bright field image; Middle column: PI (red) merged with Prestin (green); white rectangle indicated enlarged area shown in right column. Three OHC cells. (**E**): Q-RT-PCR analysis of prestin expression in kanamycin- treated mouse cochlea. Q-RT- PCR was performed using validated Taqman™ Gene Expression Assays as described in Materials and Methods. Kanamycin significantly down-regulated prestin expression. *, p<0.05; ***, p<0.01.

It has been previously shown that ototoxicity induced by AGs is a result of irreversible damage in OHC [18]. To correlate the time-dependent changes in auditory threshold shift with structural changes induced by kanamycin in OHCs, we examined structural integrity on cochlear sections from animals treated with kanamycin at the same time points as that for ABR experiments. In saline-treated mice, FITC-phalloidin staining for F-actin showed the well-defined outline of OHCs (Fig. 2Aa). After treatment with kanamycin for 7 days, there was no apparent loss of OHC (Fig. 2Ab), whereas continued injections resulted in some initial death of OHC after 14 days (Fig. 2Ac). 21 days after kanamycin administration, normal structure of basilar membrane collapsed, almost all OHCs disappeared, and the space at which OHCs were located was filled with scarred structure (Fig. 2Ad). These results are consistent with the time-dependent functional deficits determined by the ABR measurements.

### Kanamycin induces apoptosis and downregulates prestin expression in OHC

Apoptosis-related mechanisms have been proposed to be the pathophysiological basis of AGs-induced ototoxicity [4], [19]. To examine the effect of kanamycin on apoptosis in the cochleae, we stained the cochlear sections with TUNEL. In agreement with the functional and morphological result, we did not observe any dying cells in saline controls or after 7 days of kanamycin-treatment (Fig. 2Ba&b). After 14 days of drug treatment, some TUNEL reactive cells were observed in OHCs (Fig. 2Bc). In contrast, 21 days of consecutive treatment produced substantial amount of TUNEL-positive cells in OHCs (Fig. 2Bd). Of note, after 21 days of kanamycin treatment, besides OHCs, apoptotic signals were also observed in stria vascularis, supporting cells and spiral ganglion cells as well (data not shown). Taken together, the administration of kanamycin causes time-dependent hearing loss and pronounced damage of OHCs. This chronic detrimental effect is mainly attributed to kanamycin-induced apoptosis in OHCs.

The answer to the question why OHCs are more susceptible to drug-induced apoptosis compared to Inner Hair Cells (IHCs) remains elusive. Given the specific expression of prestin in OHCs, we speculated that prestin might be involved in the modulation of kanamycin-induced apoptosis in OHCs and thus examined its expression during the course of kanamycin treatment. As shown by immunostaining in Fig. 2C, prestin-positive staining in the cross section of OHCs was well organized with three rows of prestin-positive cells in the basilar membrane in control (Fig. 2Ca); however, prestin immuoreactive signal was lost in the severely destroyed OHCs after 21 days of treatment (Fig. 2Cd). Further studies on paraffin sections of the cochlea showed that in the control organ of corti, prestin was localized at the basolateral plasma membrane of three OHCs in an orderly manner (Fig. 2Da). However, kanamycin administration markedly altered the expression of prestin in a time-dependent manner. At day 21, prestin immunoreactivity was severely deranged (Fig. 2Dd).

We also analyzed time-dependent prestin mRNA expression in the organ of corti with or without kanamycin treatment by Q*-PCR*. As shown in Figure 2E, a time-dependent reduction in prestin mRNA levels was observed in response to kanamycin treatment compared to the untreated control. Of particular importance, we noted a significant reduction in prestin mRNA level 7 days after drug administration which preceded the observed structural damage of OHCs (Fig. 2A), indicating that the kanamycin-induced down-regulation of prestin expression was not secondary to OHC loss, at least not before day 14. The finding that kanamycin suppresses the expression of prestin, which is vital for hearing amplification in mammals, prior to its damaging action on OHCs may explain the clinical observation that the AGs-induced ototoxicity commonly starts with hearing threshold shift and followed by irreversible hearing loss in patients with chronic aminoglycoside treatment.

### Overexpression of prestin in Chinese Hamster Ovary (CHO) cells enhances kanamycin-induced apoptosis

**Figure 3:**
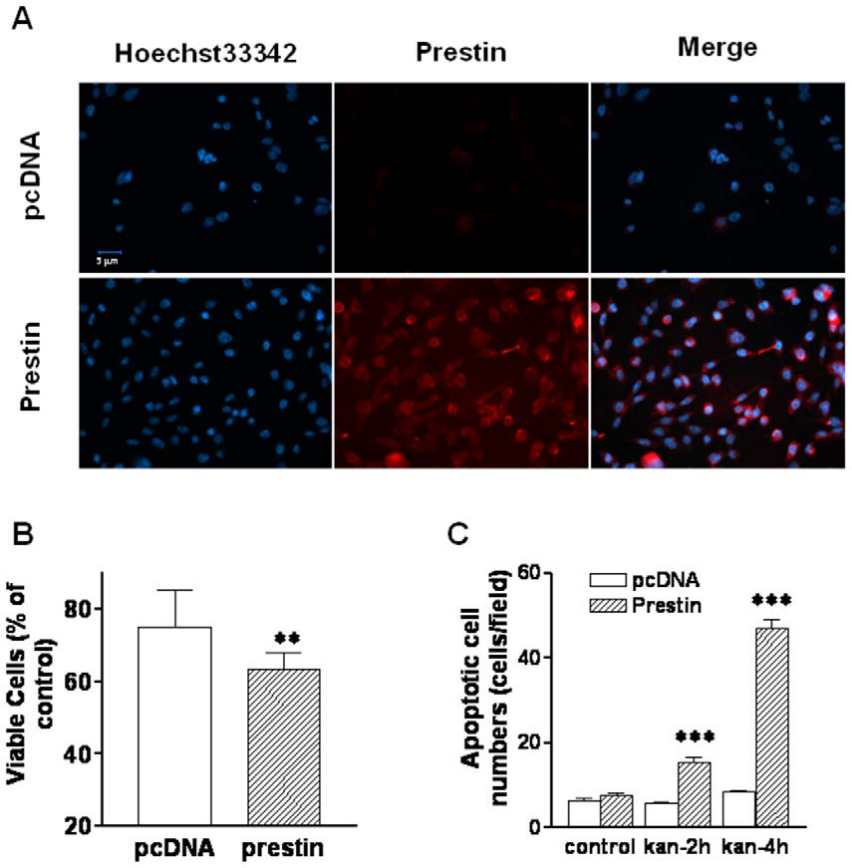
Overexpression of prestin in CHO cells enhances kanamycin-induced apoptosis. pcDNA3.1(-)-prestin or control vector were transfected into CHO cells with lipofectamine as described in Materials and Methods and the cells were treated with 100 µM kanamycin for the time indicated. (**A**): immunofluorescence staining of prestin in CHO cells showing >90% of the cells expressing prestin. (**B**): Control and prestin transfected cells were treated with 100 µM kanamycin for 2 h, and analyzed by MTS assay as described in Materials and Methods. Y axis indicates the percentage of viable cells relative to untreated cells. *, p<0.05. (**C**): Quantification of TUNEL positive cells in control and prestin-transfected cells treated with 100 µM kanamycin for 2 h or 4 h. ***, p<0.01.

Does prestin play a role in the kanamycin-induced apoptosis? If yes, through what mechanism? To answer these questions and taking into the consideration that very limited number of OHCs could be isolated from the cochlea, we transfected CHO cells, which do not express endogenous prestin, with prestin and compared their response to kanamycin to that in CHO cells transfected with empty vector. After transfection, over 90% of CHO cells expressed prestin, which was localized predominantly at the plasma membrane (Fig. 3A). MTS assay results showed that treatment with 100 µM Kanamycin for 2 h significantly decreased number of viable cells in prestin-expressing CHO cells compared with the vector control (Fig. 3B). Further TUNEL assay showed that while kanamycin did not induce significant apoptosis in control-vector transfected CHO cells, the forced expression of prestin significantly increased the number of apoptotic cells in kanamycin-treated CHO cells (Fig. 3C), indicating a predominant role of prestin in the kanamycin-induced apoptosis.

### Prestin-expressing CHO cells exhibit anion transporting capacity

**Figure 4:**
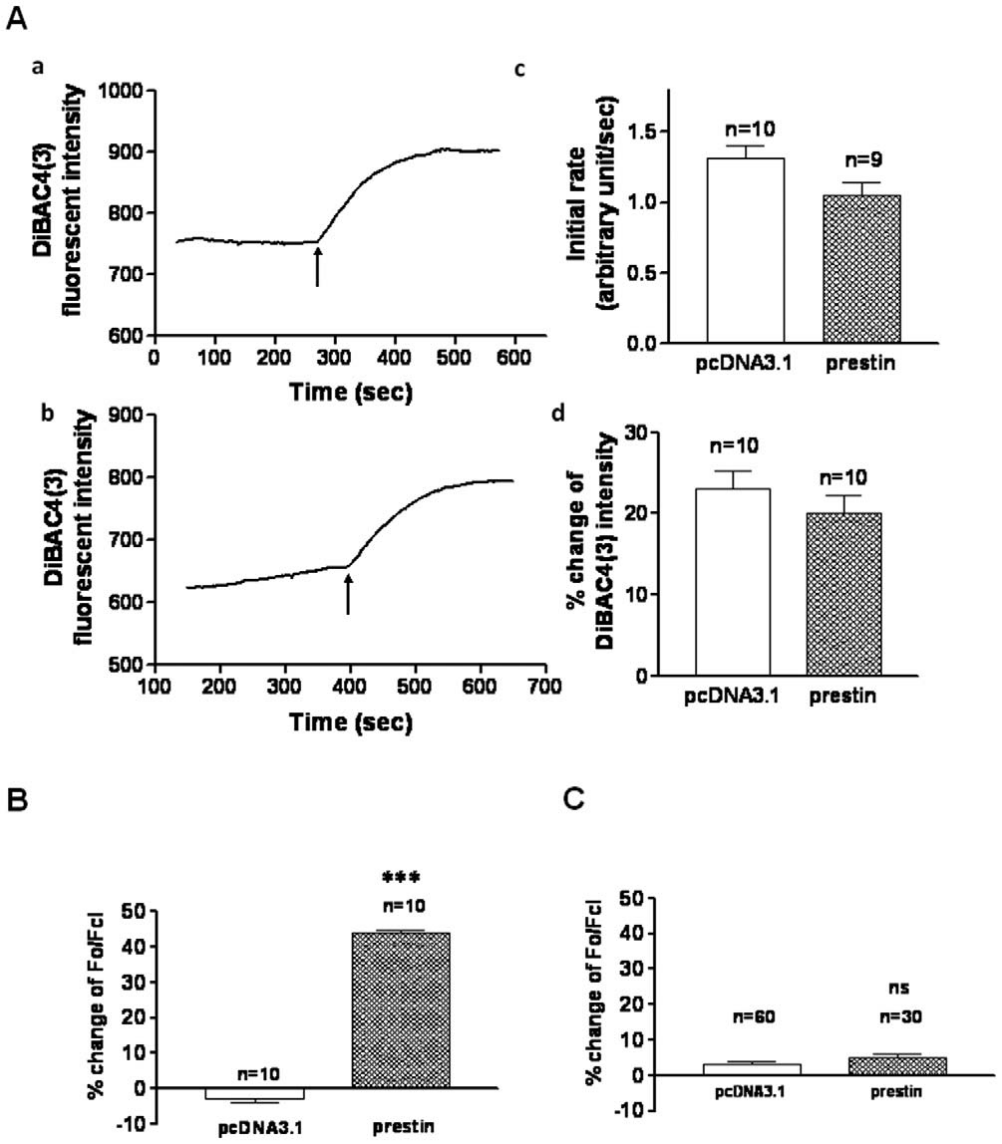
Prestin-expressing CHO cells exhibit anion transporting capacity. Control or prestin transfected cells were cultured on cover slides for 24 h, and incubated with Krebs-Henseleit (KH) medium. (**A**): Measurement of membrane potential by voltage- fluorescent dye DiBAC_4_(3). Arrows indicate the time when 100 µM kanamycin was added in the KH media. (a), Control cells; (b), prestin-transfected cells; (c), Quantitative analysis of increasing rate of membrane potential; (d), Quantitative analysis of change of membrane potential compared to the base line. (**B**): Intracellular chloride concentration was measured by MQAE dye in control or prestin transfected cells incubated in KH media. ***, p<0.01. (**C**): Intracellular chloride concentration was measured in KH media depleted of chloride. NS, no significant difference.

Having established that prestin-expressing cells are more susceptible to kanamycin-induced apoptosis, we next sought to investigate possible mechanisms underlying this differential sensitivity to kanamycin. Since prestin is known to have both voltage-sensing and ion transporting capacities [20], [21], it may sense membrane voltage changes induced by kanamycin, a known ionophore allowing influx of cations [22], with conformational changes that facilitate anion transporting capacity of prestin. We first examined whether kanamycin could induce membrane potential changes in prestin-transfected or vector control CHO cells. As shown in Figure 4A, 100 µM kanamycin induced a rapid depolarization of membrane potential as reflected by a sharp rise in the fluorescence intensity of the voltage-sensitive dye (DiBAC_4_(3)) in both vector control and prestin-expressing CHO cells (Fig. 4Aa,b), indicating cation influx induced by kanamycin as expected. Interestingly, the change in membrane potential in the prestin-expressing CHO cells was less than that of the vector control (Fig. 4Ad). In addition, the initial rate of increase in membrane potential in the prestin-expressing CHO cells also appeared to be slower as compared to that of vector control, although there was no significant difference (Fig. 4Ac). These results suggest possible concurrent influx of anions through prestin or other channels, which counteracts with the part of effect of the kanamycin-induced cation influx on membrane potential.

Next, we set out to determine whether the prestin-expressing CHOs indeed possess the anion-transporting capacity. Using a fluorescent indicator MAQE for intracellular chloride, we compared changes in intracellular chloride concentrations in response to kanamycin challenge in vector control and prestin-expressing CHO cells. As shown in Figure 4B, kanamycin treatment (100 µM) resulted in a significant increase in intracellular chloride concentration in prestin-expressing cells compared to that in control cells, indicating that prestin is required for mediating the kanamycin-induced change in intracellular chloride concentrations. When chloride was removed from the extracellular solution, the kanamycin-induced change in intracellular chloride was abolished (Fig. 4C), indicating that the kanamycin-induced change was due to chloride influx, which might be mediated by prestin with its anion-transporting capability.

### Prestin-enhanced kanamycin-induced apoptosis depends on extracellular chloride

**Figure 5:**
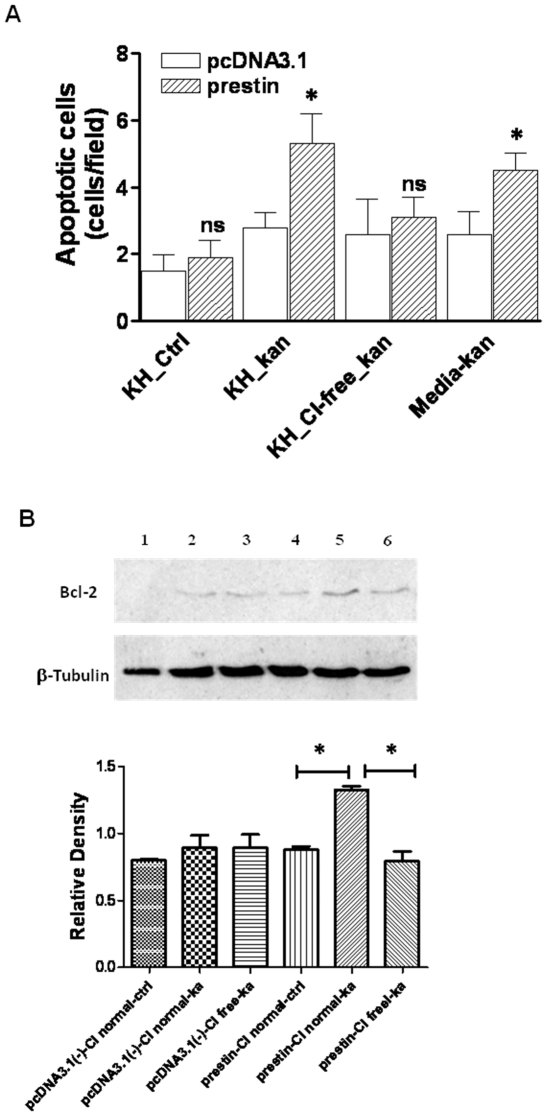
Prestin-enhanced kanamycin-induced apoptosis depends on extracellular chloride. Control or prestin-transfected CHO cells were exposed to 100 µM kanamycin for 2 h in the presence or absence of extracellular chloride in KH media. Normal culture media F-12K was used as a control. (A): TUNEL staining showed when cells were incubated in chloride-free KH solution, the kanamycin-induced effect in prestin-expressing CHO cells was abolished. *,p<0.05. (B): Western blot analysis of bcl-2 expression in control and prestin-transfected cells upon 100 µM kanamycin treatment in KH media with or without chloride for 2 h. *,p<0.05. Images are representative of 3 separate experiments.

Since anion fluxes have been implicated in regulating apoptotic activities in different cell types [23], [24], the requirement of prestin in mediating the kanamycin-induced chloride influx suggests that the anion-transporting property induced by prestin is likely to contribute to the kanamycin-induced apoptosis. In other words, the kanamycin-induced apoptosis should depend on both prestin and extracellular chloride. To test this, we treated either the control or prestin-transfected CHO cells with kanamycin in the presence or absence of extracellular chloride in Krebs-Henseleit (KH) solution, the ion composition of which could be manually manipulated. As shown in Fig. 5, forced expression of prestin significantly potentiated kanamycin-induced apoptosis as detected by TUNEL assay compared with the prestin-lacking control when cells were incubated in the chloride-containing KH solution, which was similar to that when cells were incubated in chloride-containing culture media (Fig. 5A). When cells were incubated in chloride-free KH solution, the kanamycin-induced effect in prestin-expressing CHO cells was abolished, indicating that the kanamycin-induced apoptosis depended on extracellular chloride. Thus, it appears that the anion-transporting capacity induced by prestin is involved in mediating the enhancement effect of prestin on the kanamycin-induced apoptosis.

Changes of chloride conductance have been suggested to correlate closely with the onset of apoptosis, through interaction with Bcl-2 family of proteins, both *in vitro* and *in vivo* [25], [26]. To explore the molecular mechanism underlying prestin-mediated apoptosis, both control and prestin-transfected CHO cells treated with kanamycin in the presence or absence of chloride were evaluated by western blot for relative expression of Bcl-2. As shown in Figure 5B, kanamycin significantly upregulated Bcl-2 expression in prestin- expressing CHO cells, the effect of which was completely abrogated in chloride-free solution. In contrast, there was no significant difference of Bcl-2 expression in control CHO cells.

## Discussion

The results presented here have demonstrated, for the first time, that prestin is able to act as a mediator of antibiotic-induced apoptosis, the effect of which is probably coupled to its anion-transporting capacity. While prestin has been identified as the membrane-based motor protein specific to cochlear outer hair cells and thought to be mainly responsible for active mechanical amplification in mammalian ears [16], [27], [28], a recent study revealed its ion-transporting capacity, which is independent of its voltage-sensing capacity[17]. However, the physiological significance of this ion transporting function has not been investigated. In the current study, it is clearly shown that the kanamycin-induced apoptosis in CHOs is greatly enhanced by prestin and that the prestin-enhanced effect on apoptosis depends on extracellular chloride. This suggests that chloride influx, which has been reported to be involved in regulating cellular apoptotic activities, may be involved in mediating the apoptosis-enhancing effect of prestin. This could be due to the voltage-sensing capacity of prestin that could sense the influx of cations through ionophore kanamycin, and result in conformational change that facilitates chloride transport. Alternatively, the exogenous expression of prestin may activate endogenous chloride uptake pathway(s) upon kanamycin treatment. However, this possibility and detailed mechanism remain to be investigated. Nevertheless, the results of the present study suggest that apart from generating electromotility in the mammalian cochlear, prestin is very likely to play an important role, either directly or indirectly, in transuding apoptotic signals in response to extracellular stimuli, such as antibiotics.

A growing number of evidence suggests that anions, particularly chloride, play an important role during the cell death process. Early studies on ions and apoptosis showed the activation of an outwardly rectifying chloride current (ORCC) upon CD95 receptor stimulation in Jurkat T-cells [29]. Additionally, a swelling-induced chloride current, which could be inhibited with NPPB or lanthanum, was observed in Xenopus oocytes upon treatment with pro-apoptotic sphingolipids, suggesting potential involvement of the movement of chloride in the process of apoptosis. Recently, more Cl^-^ channels have been implicated in the regulation of apoptosis in a variety of cell types (1–3)]. Among the proposed mechanisms accounting for the proapoptotic effect of anion channels, cellular acidification due to anion influx has been demonstrated to trigger mitochondria apoptotic cascade involving Bcl-2. In the present study, kanamycin is demonstrated to induce an influx of chloride and apoptosis in cells transfected with prestin. This response can be completely abrogated when chloride was removed from the extracellular media, indicating that the prestin-mediated apoptosis depends on chloride influx. Moreover, that prestin-mediated apoptosis is associated with an increase in antiapoptotic protein bcl-2, implying possible activation of intrinsic mitochondria cascade subsequent to prestin-mediated chloride influx.

The presently demonstrated apoptosis-enhancing effect of prestin, together with its well-established OHC-specific expression, may explain the observation that outer hair cells are more vulnerable to antibiotic administration compared with other cells in the cochlear. An important question that logically follows would be why prestin, the crucial protein responsible for amplification of sounds, is involved in the process of apoptosis in OHCs, the consequence of which will result in permanent hearing damage. What is the physiological significance of this seemingly paradoxical role of prestin in OHCs? Of particular interest, in our mouse model, we noticed, upon kanamycin treatment, a time-dependent down-regulation of prestin transcript prior to detectable apoptotic cell death observed in OHCs which becomes obvious on day 14. Considering its crucial role in mechanical amplification of sounds, one might expect that the reduced expression of prestin could lead to compromised hearing ability. Indeed, subsequent to a significant downregulation of prestin mRNA and disordered distribution of its protein observed on day 7, the onset of auditory threshold shift appeared around day 10, which was also prior to detectable apoptosis and deformation of the cochlea was observed after day 14. Subsequently, long term exposure to kanamycin also induced apoptosis in other cochlear cells including spiral ganglion cells and stria vascularis cells, which is expected to result in permanent and irreversible hearing loss, as indicated by a significant shift in auditory thresholds in almost all frequencies tested in our animal model. Thus, the observed down-regulation of prestin during early exposure to kanamycin appears to serve two critically important purposes, to prevent or reduce apoptosis in OHCs and to inform of hazardous environment with compromised hearing sensitivity before severe or permanent hearing damage may occur. The time window between prestin downregulation and ultimate cell damage raises the possibility to reverse aminoglycoside-induced ototoxicity at this stage in clinical practice. In fact, the transient hearing lose in patients induced by aminoglycosides can be reversed after withdrawal of drug treatment at early stage of ototoxicity[30]. Although it remains to be elucidated how prestin expression may be suppressed by kanamycin, it is clear that such suppressive effect could be one of the oto-protective mechanisms of OHCs in response to the hostile exposure.

In closing, our study has demonstrated a previously undefined role of prestin in regulating apoptosis in OHCs. Downregulation of prestin, with the coupling of its well-established capacity in sound amplification and its newly defined role in apoptosis, may serve as an important and sensitive protective mechanism against ototoxic substances. The present findings also shed new light to our understanding of the cellular process and mechanism underlying the early stage of antibiotic-induced ototoxicity. It remains to be elucidated whether prestin is involved in the final stage of antibiotics-induced cell death or other cell death mechanisms may also be involved leading to the final and permanent damage of OHCs.

## Materials and Methods

### Animal model of ototoxicity

Young adult male BALB/c mice (4 weeks old) were used for animal model of kanamycin induced ototoxicity. Mice were approved by the University Committee on Use and Care of Animals at the Chinese University of Hong Kong. *In vivo* studies were performed with the approval of the Animal Experimentation Ethics Committee of The Chinese University of Hong Kong. For ototoxic deafening, mice were divided into saline control and kanamycin treatment groups. Ten mice per group (ka-3d, ka-7d, ka-10d, ka-14d, ka-21d) were subcutaneously injected kanamycin sulfate (Ameresco, 0408-100G, USA, 750 mg/kg) twice daily for the time indicated. The development of ototoxicity was evaluated using auditory brainstem response (ABR) [18]. The animals were sacrificed 5 days after termination of the treatment and the cochleae were removed for immunocytochemistry or the extraction of total cochlear RNA.

### Cell Culture and transfection

CHO-K1 (ATCC, CCL-61) cells were maintained in F-12k culture medium (Kaighn's Modification of Ham's F-12 Medium, ATCC, 30-2004) with 10% Fetal Bovine Serum (Gibco, 10270-106) at 37°C in 95% O_2_/5% CO_2_. For transfection, CHO-K1 cells cultured in 60 mm dish for 24 h were transfected with 5 ug of pcDNA3.1(-)-Prestin or control vector (Provided by Prof. Jing Zheng at Auditory Physiology Laboratory, Department of Communication Sciences and Disorders, Northwestern University) in 10 µl of Lipofectamine (Invitrogen, 18324–020). 48 hours after transfection, cells were diluted and selected with fresh media containing 1 mg/ml G418 (neomycin) for one week, and survived cells were pooled and propagated for further analysis. For kanamycin treatment, control or prestin transfected cells were washed with PBS and incubated with 100 µM kanamycin for time indicated.

### Tissue preparation and Immunostaining

For immunohistochemistry, the mice treated with kanamycin were anaesthetized with CO_2_, and the cochlea were subsequently dissected and perfused with 4% paraformaldehyde in PBS for a while, and fixed in the same fixation fluid for overnight at 4°C. The specimens were then washed with PBS and decalcified with 4% EDTA in PBS (pH 7.4) for 3–5 days at 4°C. Decalcified cochlea specimens were then embedded in paraffin, cut into sections (5 µm), and placed on coated slides. For immunohistochemistry, sections were stained for overnight at 4°C with prestin polyclonal rabbit antibody (1∶70 diluted, Santa Cruz, sc30163, USA) using the microwave antigen retrieval technique. After washing, sections were stained with the secondary antibodies with peroxidase-conjugated anti-rabbit followed by rabbit peroxidase anti-peroxidase (Dako Corporation, CA) for 1 hour at room temperature. After being washed, color was developed with 3, 3-diaminobenzidine and counterstained with hematoxylin. An isotype-matched IgG was used as a negative control throughout the study.

For immunofluorescence, the specimens were rinsed with PBS and the bone surrounding the organ of Corti removed. Subsequently, the tissues were fixed in 4% paraformaldehyde for 1 h at room temperature, followed by permeabilization with 0.3% Triton X-100 for 1.5 h. After a rinsing in PBS, the tissues were incubated for 2 h in blocking solution containing 2% bovine serum albumin and 5% normal goat serum in PBS. Tissues were rinsed in PBS and incubated with prestin polyclonal rabbit antibody (1∶70, Santa Cruz, sc30163, USA) or FITC-conjugated Phalloidin (1∶100 sigma, p5282, USA). Tissues were rinsed in PBS for 10 min, and incubated for 30 min with a secondary antibody (1∶200 diluted, goat anti-rabbit antibody conjugated Rhodamine, Santa Cruz, sc2091, USA). After a 10 min PBS rinse, the basilar membrane was separated and mounted on the slides in anti-fading mounting medium and photographed with the laser confocal microscope (Zeiss).

### Isolation of cochlear total RNA and RT-PCR (Q-PCR)

Total RNA was isolated from mice cochlea by using TRIzol reagent (Invitrogen) following the protocol provided by the manufacturer. Total RNA (5 µg) was reverse transcribed using M-MLV Reverse Transcriptase (USB, GE Healthcare) in a 20-µl reaction and 2 µl of reverse-transcription product was amplified by PCR using standard protocols. Prestin TaqMan primer and probes were obtained from Applied Biosystems (Mm01167265_m1). A 96-well plate was used for the PCR reactions. Assays were performed in triplicate on an Applied Biosystems 7500Fast Real-Time PCR System and average Ct values normalized relative to expression of mouse-slc26a5.

### MTS assay

Control or prestin transfected cells were plated in triplicate wells at a density of 5×10^3^cells/well in 96 flat-bottomed plates, allowed to attach overnight, and then 100 µM kanamycin added for 2 h. Cell viability following drug treatment was assessed using the CellTiter-GIo Luminescent Cell Viability Assay (Promega, Madison, MI).

### Western Blot

Whole cell lysates were analyzed by western blot using standard procedures. Primary antibodis used were: anti-bcl-2 (1∶500) (Santa Cruz Biotech), anti-tubulin (1∶1000) (Santa Cruz Biotech).

### Membrane potential measurement by voltage-sensitive fluorescent dye

The measurement of membrane potential changes by DiBAC_4_(3) [Bis-(1,3-dibutylbarbituric acid)-trimethine oxonol] dye was performed with excitation maxima at approximately 490 nm. Briefly, cells were cultured on cover slides for 24 h, and incubated with Krebs-Henseleit (KH) medium adjust to 290mOsm with Glucose, balanced pH with 95% O_2_, 5% CO_2_ mixed gas) contained 1 µM/ml DiBAC_4_(3) for 20 min at room temperature in darkness. Experiments were carried out in the constant presence of DiBAC_4_(3) dye. The fluorescence emission was collected at 535 nm. Depolarization results in a increasing in fluorescence intensity, whereas hypo-polarization causes decreasing fluorescence intensity in cells [31].

### Intracellular chloride measurement by MQAE dye

Cells were cultured on round cover slides for 24 h. Before measurement, cells were incubated in 2 ml KH solution contained 10 mM MQAE dye [N-(6-methoxyquinolyl)-acetoethyl ester] for 30 min at 37°C in darkness. Cells were then incubated in fresh KH solution without MQAE dye, the intracellular fluorescence intensities were measured at 460 nm.

### Terminal deoxynucleotidyl transferase-mediated dUTP nick end labeling

DNA fragmentation was detected by the TUNEL technique, which detects DNA fragmentation during apoptosis. For tissues, the specimens were prepared as described above. For CHO-K1 cells, cells transfected with prestin or pcDNA3.1(-) were cultured on cover slides for 24 h and treated with 100 µM kanamycin for 2–4 h. Cells were then fixed with methanol and acetone (1∶1 mixed) for 15 min at 4°C, washed with PBS, and permeabilized with 0.3% Triton X-100 for 20 min. Tissues or cells were then incubated with the TUNEL assay reaction mixture (mixed enzyme solution with label solution at dilution 1∶9, In Situ Cell Death Detection Kit, Roche) at 37°C, in darkness for 60 min, followed by counterstained with Propidium Iodide (PI) or Hoechest 33342 for 10 min. After thoroughly washing with PBS, the samples were mounted in anti-fading mounting medium.

### Statistical Analysis

The data were processed using Prism 3.0 software. Values are reported as means ± S.D. if not indicated otherwise. Comparisons among values for all groups were performed by one-way ANOVA or student t test. A “P” value of less than 0.05 was considered statistically significant.
